# The contemporary immunoassays for HIV diagnosis: a concise overview

**DOI:** 10.2478/abm-2023-0038

**Published:** 2023-08-07

**Authors:** Abdullah, Misbahud Din, Abdul Waris, Muddasir Khan, Sajjad Ali, Riaz Muhammad, Muhammad Salman

**Affiliations:** Department of Health and Biological Sciences, Abasyn University Peshawar, Peshawar 25000, Pakistan; Department of Biotechnology, Quaid-i-Azam University Islamabad, Islamabad 45320, Pakistan; Department of Biomedical Sciences, City University of Hong Kong, Shezhen 518057, Hong Kong SAR; Centre of Biotechnology and Microbiology, University of Peshawar, Peshawar 25120, Pakistan; Department of Zoology, University of Buner, Buner 19281, Pakistan; Department of Zoology, Government Degree College Lakarai, Mohmand 24651, Pakistan; Department of Veterinary Microbiology, Faculty of Veterinary Science, Chulalongkorn University, Bangkok 10330, Thailand

**Keywords:** antibodies, core protein p24, diagnosis, HIV, immunoassay

## Abstract

Recent advances in human immunodeficiency virus (HIV) diagnostics have improved the management of disease progression significantly, which have also boosted the efficacy of antiviral therapies. The detection of HIV at the earliest is very important. A highly recognized and effective virological biomarker for acute HIV infections is p24 antigen. This brief overview is based on advances of HIV diagnosis while focusing on the latest HIV testing technologies including HIV-specific antigens detecting assays of both anti-HIV antibodies and p24 antigen. In addition to other emerging molecular diagnostics for acute HIV infection, the utilization of p24 antigen has been summarized. Moreover, it has been explained how these immunoassays have reduced the window period for detection of HIV in the acute stage of infection.

Viral diseases are considered as a major public health concern worldwide. Either these viruses are blood-borne for example, hepatitis B virus (HBV), hepatitis C virus (HCV), and human immunodeficiency virus (HIV), or from other sources, for example, SARS-CoV-2, and monkeypox virus, but there is a need of rapid diagnosis, treatment, and prevention. However, HIV is a causative agent of acquired immunodeficiency syndrome (AIDS), and could be categorized by the appearance of opportunistic infections and a long period of clinical latency [1, 2, 3, 4, 5].

HIV replicate in T lymphocytes while AIDS is characterized by the loss of helper T cells that express the CD4+ surface proteins [6]. The CD4+ cells count (<200/mm^3^) could be used as a marker for disease severity and the risk of opportunistic infections [7]. In the early phase of HIV, the virus is detectable in the blood via cDNA, RNA, and p24 antigen. The presence of indeterminate HIV antibodies is usually detected in the acute stage of infection. Detection of the virus in the acute stage is of significant importance concerning various issues, for example, public health, clinical diagnosis, and early treatment [7]. Patients with acute stage of the disease are at the highest risk of secondary transmission as they contain high viral load [8].

Since the emergence of the HIV pandemic, the immunoassays for HIV detection have been continuously developed to detect the HIV-specific antibodies. The third-generation assays, in contrast to the first and second generation, detect immunoglobulin G (IgG) and immunoglobulin M (IgM) at notable advancement in HIV diagnosis [9, 10, 11]. HIV-specific antibodies are not produced during acute infection; therefore, it is a major limitation in immunoassays. Various attempts have been made for the detection of HIV during the acute phase of infection, for example, the detection of HIV-RNA that contribute significantly to HIV diagnosis [12, 13, 14, 15]. The RNA-based detection has some limitation related to effectiveness, is time-consuming, and needs expert personnel for operation and result interpretation; therefore, the fourth-generation immunoassay was developed for detection of acute infection to utilize the antigen–antibodies combination [16, 17]. The fourth-generation assay is less expensive, easy to perform, and is an automated assay. This assay simultaneously detects antibodies and a capture immunoassay for detection of the most abundant protein of HIV virions, that is, p24. This latest assay (ADVIA, Centaur HIV Ag/Ab) can differentiate p24 antigens and antibodies in the serum and therefore have reduced the window period. Among them, some of the assays can be used to detect HIV from serum, plasma, and even from whole blood samples in a very limited time, that is, 20 min. Some reports rated to fourth-generation immunoassays have claimed a sensitivity of 88% and above, of the latest assays which detect p24 antigen [18]. Other studies have reported more advanced fourth-generation immunoassays with the highest specificity and sensitivity. The accurate diagnosis of HIV is dependent on the analytical sensitivity of the test to p24 antigen and the efficiency to detect the diversity in HIV strains. The rapid evolution of HIV has resulted in the production of various subtypes and recombinant viruses [19, 20].

Currently, HIV-1 is categorized into various groups like M, O, N, and P. Among them, group M is the most prevalent group of HIV-1, which has been further classified into nine subtypes (A, B, C, D, F, G, H, J, and K) and more than 75 circulating recombinant forms (CRFs) and several unique recombinant forms (URFs) [19]. This extensive diversity of HIV poses challenges for reliable detection assays maintenance in routine diagnosis. Several cases of HIV strains have been reported to be escaping in fourth-generation assays [11, 21]. A recent study reported that HIV diversity impacted the sensitivity of fourth-generation assays in the early HIV infection [22]. Of note, highly active antiretroviral therapy (HAART) is the treatment regimen, particularly the cocktail of three or more anti-retroviral drugs. The combination of these drugs inhibits replication of HIV-1, and by action reduces the viral load, reducing the morbidity and mortality of patients, and boosts the immune system. It is noted that when HAART is interrupted, HIV-1 antigen persists and viral rebound may occur, which ultimately increases the level of CD4+ cells and p24 antigen [23].

The current review was aimed to present an overview based on advances in HIV diagnosis while focusing on the latest HIV testing technologies, including HIV-specific antigens and antibodies detecting assays. The review also focuses on p24 antigen assays and other emerging molecular diagnostics, and these immunoassays have reduced the window period for detection of HIV in the acute stage of infection.

## HIV Ab detection assays

HIV-1 and HIV-2 have various available commercial diagnostic assays for screening blood, infection detection, and analysis of the disease continuation in infected individuals since 1986. These assays could be classified into four major groups: those used to detect anti-HIV antibodies, p24 antigen, viral nucleic acid (detection or quantification), and T-lymphocytes count estimation [24].

The most particular and common immunoassay utilized for HIV antigen-antibody detection is the enzyme-linked immunosorbent assay (ELISA). The technique has developed from tests of viral-lysate-based IgG to first-generation tests (window period, 40–50 days), second-generation tests (30–35 days) containing synthetic peptide antigens, and recombinant third-generation tests (20 days) which detect IgG, IgM, and fourth-generation HIV tests [24]. Certain limitations noted in the above-mentioned tests were, first-generation tests were less efficient in specificity compared with sensitivity, second-generation assays were noted to be excellent in regard to specificity and sensitivity but the maximum cross-reactivity of Abs was prominent with group M subtypes, and third-generation assays were used in detection of the early stages of infection. Moreover, these assays are still used in developing countries for detection of vaccine-induced Abs as well as infection acquired by HIV individuals [3].

Later in the 1990s, companies made fourth-generation tests for HIV antigen–antibody detection in a combined slot. These techniques were based on the principles of ELISA and chemiluminescence tests. The test negative gap was narrowed by these tests into minimum time (2–18 days). The limitation associated with these tests included only a single result of detection of antigen–antibody interaction. These assays lack to evaluate generally and geographically circulating subtypes of HIVI/II. The Food and drug administration (FDA) approved the Abbot Architect method of these procedures in August 2010, with 99.94% sensitivity and 99.95% specificity of repeat testing in a study population [25]. Similarly, in 2020, the FDA approved G4 ELISA of antibody rapid test, the research team evaluated G4 ELISA which displayed 100% sensitivity for HIV-1 and 98.18% for HIV-2 while specificity was 100% [26]. According to a study, fourth-generation ELISA (Abbot Architect) tests are more specific than third generation. The study evaluated 7516 subjects for third-generation ELISA with 46% positive predictive value (PPV), and 70% PPV was noted for fourth generation (*n* = 7802) [27]. The frequently used fourth-generation assays are presented in **Table 1**.

**Table 1.** Fourth-generation tests for HIV antigen-antibody

**Test**	**Manufacturer**	**Specificity (%)**	**References**
ADVIA Centaur HIV Ag/Ab Combo	Siemens	99.74	[28]
AxSYM HIV Ag/Ab Combo	Abbott Laboratories	99.9	[29]
ARCHITECT HIV Combo	Abbott Laboratories	99.4	[30]
VIDAS HIV DUO Ultra	BioMerieux	99.84	[31]
Cobas Core HIV Combi EIA	Roche Diagnostics	99.3	[30]
Elecsys HIV Combi	Roche Diagnostics	99.8	[32]
HIV Combi Modular E170	Roche Diagnostics	99.7	[30]
Enzygnost HIV Integral	Dade Behring	99.6	[30]
Genscreen Ag/Ab HIV Ultra	Bio Rad	99.7	[30]
Genscreen PlusHIV Ag/Ab	Bio Rad	99.9	[28]
Vironostika HIV Uniform II Ag/Ab	Organon Teknika	97.21	[30]
Liaison XL Murex HIV Ab/Ag	DiaSorin	98.5	[33]

EIA, HIV, human immunodeficiency virus.

## Confirmation of HIV by immunoblot assays

For the diagnosis of HIV, the serological testing of fourth-generation assay must be confirmed via secondary confirmatory method. The most frequently used secondary confirmatory method for HIV infection could be Western blots (WBs) or immunoblot assays (IBAs) using different HIV recombinant proteins, that is, p17, p24, p31, gp41, gp120, 124. In order to reduce the window time of WBs for primary screening (ELISA), WHO recommends recombinant immunoblot assay (RIBA), used as secondary confirmatory method for the detection of HIV or HCV in the early phase of infection, when the primary screening is imprecise or indeterminate. According to authors, IBAs has the highest positivity rate than WB assay. They compared two IBAs, INNO-LIA HIV 1/2, and Geenius HIV 1/2 with WB [34]. Another study from Wuhan China, also investigated HIV-1 11068 positive cases of two fourth-generation kits of ELISA on WB and RIBA (WANTAI, Biopharm, China). The indeterminate cases of RIBA were significantly less than WB [35]. In contrast, RIBA is mostly used for the detection of HCV detection than HIV [36, 37].

## Fifth-generation HIV tests

In 2015, Bio-Rad developed BioPlex 2200 HIV Ag\Ab screening test method as diagnostic assay. This multiplex analysis method was called fifth-generation HIV detection assays, later approved by the FDA. The fifth-generation HIV immunoassay detects HIV-1 p24 Ag, HIV-1 Ab (groups M and O), and HIV-2 Ab and offers discrete single signal to cutoff (S/CO) ratio data. A positive screen is defined as S/CO ratio of 1 or more on any of the three components of the BioPlex assay, with all BioPlex positive screens being repeated before advancing to confirmatory testing. Like fourth-generation tests, it provides screening of both antigen–antibody but separately of both analytes. The BioPlex fifth-generation assay results were evaluated similarly for both sensitivity and specificity [27]. Similarly, LIAISON HIV Ag-Ab immunoassay has also proved excellent diagnostic sensitivity in the detection of HIV in the early phase of infection with the least of window timing (2–5 days). According to a previously conducted study, the diagnostic performance of LIAISON was evaluated for HBV, HCV, and HIV infection along with increasing S/CO for subsequent confirmation of HIV-1 via LIAISON. Among all HIV positive (*n* = 229) patients, 1.6% were reactive with 141 (62.1%) confirmed. The increasing ratio of S/CO to 4 automatically increases PPV without uncertainty in sensitivity [38].

## Rapid HIV assays

Rapid HIV assays are card-based procedures that have mainly gone through first- and third-generation assays as a basic screening test. These tests are developed for whole blood, serum, and oral fluid. In 2012, FDA approved a newly developed point of care method named “OraQuick” for home testing, with effective testing capabilities [39]. This test is different from other conventional tests, and can be performed without any laborious assistance at home within 20 min. Similarly, Geenius semi-automated and differentiation assay for HIV-1 and HIV-2 is a rapid assay approved (FDA) and can only be used as supplemental technique in the fourth-generation algorithm, but not as a screening test. It has been reported that Geenius has 100% sensitivity and 96% specificity [38]. Presently, there is a combo of fourth- and fifth-generation rapid assays which is used for the detection of HIV antigen and antibodies but does not distinguish between antibodies of HIV-1 and HIV-2. It has been reported in a study that it has 100% of specificity 88.2% sensitivity [39].

## Nucleic acid-based HIV-detection

Since 1995, several nucleic acid-based detection tests have been established for the estimation of viral RNA (HIV) in blood plasma. Three main techniques used for the detection of HIV nucleic acid are reverse transcriptase polymerase chain reaction (RT-PCR), branched chain DNA (bDNA,) and nucleic acid sequence-based amplification (NASBA). The viral RNA (HIV-1) detection was achieved after amplification of products with labeled probe, which was finally detected by immunoassay or chemiluminesence technology [40]. Currently, real-time techniques (PCR-based HIV RNA assays) constantly detecting fluorescence emanated during every cycle of PCR [41]. HIV-1 RNA estimation is achieved during the first phase of PCR, as compared with conventional PCR or other technologies and provide more reliable results. Owing to new developed assays, the detection number has been improved much higher than old technologies (~50 copies/mL) [42]. According to a current study, a new combo of two detection techniques, including PCR (gene amplification), followed by ELISA (NAT-ELISA), has been developed by a group of scientists for the detection of HIV-1,2 and other blood-borne viruses (HBV & HCV), in their window timing. The WHO standard protocol for PCR amplified products were followed; 321bp of HIV-1, 291bp (HIV-2), 229bp of HBV, and 105bp of HCV. The limit of detection (LOD) 95% and LOD 97.7% of HIV-1, HIV-2 HBV, and HCV were 13 IU/mL, 6 IU/mL, 15 IU/mL and 11 IU/mL, and 15 IU/mL, 7 IU/mL, 17 IU/mL, and 12 IU/mL individually. The overall sensitivity and specificity of NAT-ELISA assay was noted at 97.4% and 99.4%, respectively. The newly developed assay (NAT-ELISA) suggests overcoming complications of previously used methods such as cost-effectiveness, handling, and compromising sensitivity for high performance [43].

## Nano-technology in HIV diagnosis

Precise, accurate, and minute technologies are required to characterize, visualize, and manipulate molecular targets at the nanoscale [44]. Nanotechnology refers to the fabrication, design, characterization, and application of devices and materials whose size is 1–100 nm from at least one dimension. Nanotubes, nanofibers, nanosheets, nanorods, nanoplates, nanodots, nanowires, and nanobelts have been defined based on this description [24]. The rapid developments in nanoscience and nanotechnology have driven innovations in all fields of science, including biosensors, where cutting-edge research is carried out to develop the diagnosis of various diseases [45]. Nano-sized materials are opening new opportunities in the area of sensing by adding more value to existing sensing technologies [46]. They increase the loading of recognition elements on the platforms, signal amplification, speed of response, detection range, selectivity, interference, specificity, cost-effectiveness, and stability [47]. Substantially, multifarious nanomaterials play a key role in developing point of care testing and multi-modality sensing tools.

A number of direct and indirect methods have been developed for the detection and diagnosis of HIV [32]. These various approaches detect various biomarkers, including different types of HIV antibodies (HIV type 1 and HIV type 2 antibodies), viral p17 and p24 antigen, viral nucleic acid, CD4 T lymphocytes, and HIV-related enzymes [48]. Nanotechnology provides possibilities in the development of these bio-sensing assays by increasing sensitivity and specificity and detection at low viral concentrations [49, 50]. Through nanotechnology, devices with a high degree of specificity and sensitivity can be designed to allow the interaction of various cells and other components at the molecular level [51]. The nanoclusters exhibited different properties such as high surface-to-volume ratio, and optical and electrical properties that increase these biosensors' sensitivity and specificity [52]. Metal oxide and metallic nanoparticles, nanoclusters, quantum dots, and carbon nanostructures are valuable materials for developing a highly sensitive and specific biosensor for the diagnosis of HIV.

Harvey and colleagues designed and developed a nanobased approach for the rapid detection of serum-based HIV. This sensing technology eliminates preprocessing of the sample, such as purification of oligonucleotides and further amplification. In this approach, the protein interacts with the carbon nanotube surface and then hybridization occurs to detect the virus, yielding a photoluminescence response [53]. Similarly, the development of POC lateral flow assays (LFAs), termed as nano-tags that detect the HIV-1 DNA marker using gold nanoparticles and surface-enhanced Raman scattering. The LFAs are therefore highly accurate with a 100 times higher sensitivity than fluorescent or colorimetric detection method [54].

Gray and colleagues applied nanotechnology to design and develop a mobile antibody-based biochip sensor using smartphone mechanism. They reported that this biochip sensor would be precise and accurate in the diagnosis of anti-gp41 with the highest specificity and sensitivity (100%) and give the results within 1 min [55]. Other nano-based techniques that show high specificity and sensitivity in diagnosing HIV nucleic acid include a photoelectrochemical biosensor, amplified electrochemiluminescence biosensor, strand displacement amplification, and copolymer nanospheres.

## Plasmonic nanoparticles-based approach for HIV diagnosis

Localized surface plasmon resonance (LSPR) is commonly not exhibited in the bulk materials but associated with nanosized materials [56]. Surface plasmon resonance (SPR)-based diagnosis leads to alter the refractive index quantity in the electromagnetic field [57]. This approach is reliable, sensitive, and rapid and offers hugely responsive sensing to various biomolecular reactions, including viruses. An LSPR approach has been exploited to detect HIV at point of care in resource limited laboratories. For the detection of various subtypes of HIV, a nano-plasmonic-based approach has been developed. This assay has the highest accuracy (98 ± 39 copies/mL) and reliability. Gold nanoparticle-based immunochemistry is done using a whole blood sample, and there is no need for sample preprocessing to capture the agent (virus). A plate reader, usually a microplate titer, is used to quantify the transmission/absorbance to measure the captured intact virus [58].

However, the nano-based techniques to screen the biomarker of interest should be selected at a proper timeframe to confirm that the marker of interest is present. As there is a specific range of timeframe for each marker that is, HIV nucleic acid (RNA) can be detected about 10 days after infection while the antibody testing should occur 3–5 weeks following the infection.

## The use of p24 assays in early detection

**Figure 1.**
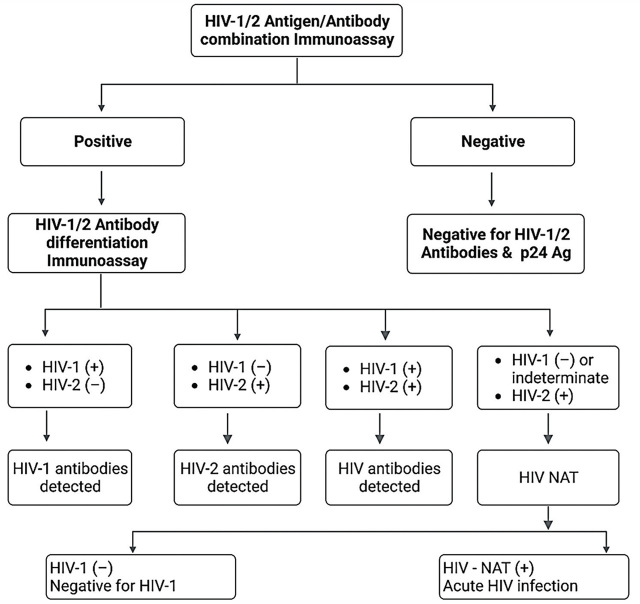
Recommended laboratory HIV testing algorithm by CDC [66]. CDC, Center for Disease Control; HIV, human immunodeficiency virus.

**Figure 2.**
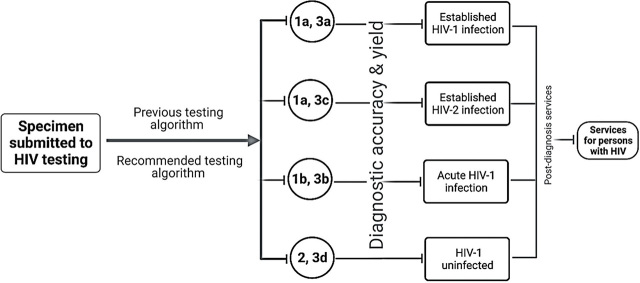
Recommended analytical framework by CDC for accurate HIV diagnosis. CDC, Center for Disease Control; HIV, human immunodeficiency virus.

**Figure 3.**
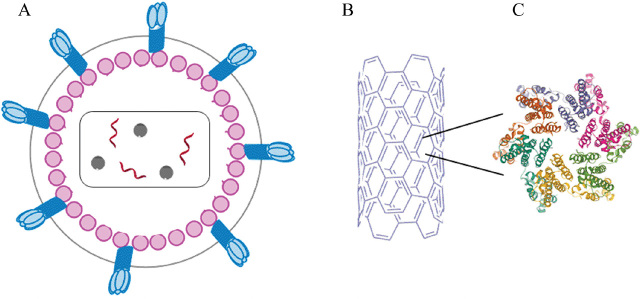
Structure of HIV and p24 antigen. **(A)** A complete virion, **(B)** capsid fullerene cone superstructure, and **(C)** monomer unit [8]. HIV, human immunodeficiency virus.

As discussed earlier, the period in which HIV is present in the body but the antibody response has not been mounted is known as acute HIV infection. Infection in the acute stage can only be detected via highly sensitive diagnostic assays that detect viral antigens [59, 60, 61]. The fourth-generation assays are recommended in early infection in adult individuals [62, 63]. The recommended laboratory HIV testing algorithm by the Center for Disease Control (CDC) along with the analytical framework for accurate detection of HIV infection is represented in **Figures 1** and **2**. In the earliest stage of infection, the HIV RNA is an important detectable viral biomarker and therefore the nucleic acid amplification test could be used for HIV detection [63]. The nucleic acid test is generally not approved as a quantitative diagnostic tool in several countries [64]. On the other hand, the p24 assays have been recommended for early HIV detection. The commercially available p24 antigen or antibodies-based assays have been reported to have higher sensitivities across the clades and recombinant forms. It has also been described that small sequence differences do alter the p24 tertiary structure and therefore the sensitivity of p24 antigen detection-based assay was not affected. Several emerging ultra-sensitive methods for detection of HIV p24 antigen are represented in **Table 2**. The FDA approved fourth-generation assays and standalone p24 assays have demonstrated increased sensitivity of next-generation p24 antigen detection platforms. The structure of HIV along with the p24 antigen is represented in **Figure 3**. Several studies have examined the properties of p24 antigen and its association with other biomarkers during HIV infection [65]. With the advanced research in virology, the next-generation technologies for intended resources limited setting could use the p24 antigen as the most effective virological target.

**Table 2.** Emerging ultra-sensitive methods for detection of HIV p24 antigen

**S. no.**	**Legend**	**Reported by**
Nanoparticles/beads based		
1	Gold†	[50]
2	Platinum-shell coated gold‡	[67]
3	Carbon§	[68]
4	Latex†	[69]
Enzyme based		
5	Urease||	[70]
6	Catalase||	[71]
7	Horseradish peroxidase||	[72]
8	Alkaline phosphatase||	[73]
9	Glucose oxidase||	[74]

† Signal enhancement method.

‡ Signal enhancement and p24 nanobody.

§ Biosensor platform.

|| Signal enhancement and biosensor platform.

HIV, human immunodeficiency virus.

## Conclusion

The testing technologies and the new-generation assays for HIV screening have been developed rapidly. The earlier diagnosis of HIV is very important in several aspects; therefore, new algorithms need to be established. The biomarkers-based diagnosis has been the main detection method for HIV diagnosis. For rapid detection, high diagnostic accuracy and earlier detection of new technologies with the use of nanotechnology are needed.

## Abbreviations:

AIDS: acquired immunodeficiency syndrome
bDNA: branched chain DNA
CDC: Center for Disease Control
CRFs: circulating recombinant forms
ELISA: enzyme-linked immunosorbent assay
FDA: food and drug administration
HAART: highly active antiretroviral therapy
HBV: hepatitis B virus
HCV: hepatitis C virus
HIV: human immunodeficiency virus
IBAs: immunoblot assays
IgG: immunoglobulin G
IgM: immunoglobulin M
LFAs: lateral flow assays
LSPR: localized surface plasmon resonance
NASBA: nucleic acid sequence-based amplification
RIBA: recombinant immunoblot assay
RT-PCR: reverse transcriptase polymerase chain reaction
SPR: surface plasmon resonance
URFs: unique recombinant forms
WB: western blot
